# Prevalence of Musculoskeletal Disorders Among Perfusion Staff in Germany

**DOI:** 10.3390/ijerph23020156

**Published:** 2026-01-27

**Authors:** Andreas Richard Greßler, Maximilian Kehmann, Claus Backhaus, Niels Hinricher

**Affiliations:** 1 Center for Ergonomics and Medical Engineering, FH Münster University of Applied Sciences, Bürgerkamp 3, 48565 Steinfurt, Germany; 2 Institute of Psychology and Ergonomics, Technische Universität Berlin, Fasanenstraße 1, 10623 Berlin, Germany

**Keywords:** musculoskeletal disorders, prevalence, perfusion, healthcare professionals, NMQ, survey, human factors in healthcare, occupational health, lower back pain

## Abstract

**Background:** Musculoskeletal disorders (MSDs) are highly prevalent among healthcare workers, particularly nurses, with reported prevalence rates ranging from 57% to 93%. Perfusionists are highly specialized healthcare professionals responsible for operating heart–lung machines during cardiac surgery. To date, the prevalence of MSDs in this professional group has not been systematically investigated. To address this gap, perfusionists in Germany were surveyed regarding MSDs. **Methods:** The German version of the Nordic Musculoskeletal Questionnaire (NMQ) was administered. Pearson’s correlation and chi-square tests were applied. **Results:** A total of 287 perfusionists (72 female, 215 male; age 42.6 ± 11.9 years, professional experience 13.5 ± 10.9 years) from 45 German cardiac centers participated. Overall, 86% reported MSDs, with the lower back (65.5%) and neck (58.9%) being the most frequently affected regions, and 4.5% to 36.6% in other body regions. Increasing age was significantly associated with a higher prevalence in six body regions, and greater professional experience was associated in five regions. Occupational risk factors previously identified in nursing are assumed to apply to perfusion practice. **Conclusions:** This study demonstrates a high prevalence of MSDs among perfusionists in Germany. These findings underscore the need for preventive measures, coping strategies, and further research to reduce work-related musculoskeletal strain among perfusionists.

## 1. Introduction

Musculoskeletal disorders (MSDs) are functional impairments of the musculoskeletal system affecting muscles, tendons, ligaments, or nerves. They are often accompanied by pain or functional limitations and have been recognized as leading causes of work incapacity worldwide [1,2,3]. MSDs are highly prevalent among healthcare professionals, particularly among nursing staff [4,5,6,7]. Previous reviews have reported annual prevalence rates in healthcare workers ranging from 57% to 93%. Commonly affected body regions include the lower back (46–84%), neck (38–71%), shoulders (33–74%), and knees (20–61%) [5,7,8,9,10].

One occupational group in which MSD prevalence has not yet been investigated is perfusion staff. Perfusionists are highly specialized healthcare professionals who operate heart–lung machines (HLMs) in the operating room (OR). HLMs supply oxygen and nutrients to organs and tissues during cardiac and vascular surgeries, temporarily replacing cardiac and pulmonary functions. They are operated and continuously monitored by perfusionists [11]. These professionals sit or stand in front of the machine throughout the surgery, which may last several hours. Furthermore, their work involves assembling single-use components and moving the heavy HLM equipment. Long working hours and high workloads in healthcare further contribute to physical strain.

Certain work characteristics are known to elevate MSD risk, including prolonged static positions, extended standing or sitting, working in twisted or bent positions, moving heavy equipment, and irregular or long work schedules [5,8,10,12,13,14].

As of early 2025, approximately 650 perfusionists were practicing in Germany [15,16]. There are an estimated 5300 perfusionists in Europe [17] and 5000 in the United States [18]. This limited workforce, combined with a broader shortage of healthcare professionals, poses significant risks to healthcare systems and, therefore, to the public. According to Turnage et al. (2017), the perfusion profession is particularly vulnerable to staffing shortages because replacing these highly specialized professionals is challenging [19]. Moreover, Lewis et al. (2016) reported that 39% of perfusionists in the United States are expected to leave their profession within a decade [20]. Therefore, retaining skilled personnel and ensuring their physical health is crucial to maintain proper healthcare.

Considering the likelihood that established MSD risk factors are present in perfusion workplaces, this study aimed to investigate the prevalence of MSDs among perfusion staff in Germany.

## 2. Materials and Methods

This multicenter, cross-sectional, observational study included perfusionists in cardiac centers across Germany. Ethical approval for the study protocol was obtained from the ethics committee of FH Münster University of Applied Sciences.

All 78 centers listed by the German Association of Perfusion were invited to participate in this study [15]. The revised and validated German version of the Nordic Musculoskeletal Questionnaire (NMQ) was used [21,22,23,24,25]. The questionnaire gathered demographic data and the 12-month prevalence of MSDs in ten body regions. Pain intensity, functional limitations and occupation-specific risk factors were not assessed. Questionnaires were distributed to participating cardiac centers and completed individually. All participants received written information regarding the context of the study and data protection procedures. Written informed consent was obtained prior to participation. The completed questionnaires were digitized and analyzed statistically using SPSS (version 29.0.1). Continuous variables are presented as means with 95% confidence intervals, standard deviations, and ranges. Categorical data is presented as frequencies and percentages. MSD prevalence was calculated for the entire study population and expressed as a percentage for each body region.

Demographic variables were tested for correlations using Pearson’s correlation coefficient. For subgroup analyses, age, body mass index (BMI), and years of experience (YoE) were categorized into four groups based on practical considerations and findings from previous studies [12,14,25,26,27,28]. Age was categorized as ≤35, 36–45, 46–55, and >55 years. This approach reflects the delayed entry of many individuals into the profession, while ten-year intervals were chosen to capture meaningful differences across career stages. BMI was classified as underweight, normal weight, overweight, or obese, according to standard definitions. YoE was grouped as ≤5, 6–10, 11–20, and >20 years, based on evidence that MSDs often emerge within the first 5 years of professional practice and stabilize in frequency over the following five and ten-year intervals [10,29,30].

An exploratory analysis was conducted to characterize the occurrence of MSDs within the cohort, providing a cross-sectional overview. The occurrence of MSDs among the different groups within the cohort was analyzed using the chi-square test. Fisher’s exact test was applied when expected cell counts were <5. The tests were performed as paired crosstab tests. Each subgroup was sequentially compared with the rest of the cohort to identify periods of significant changes in MSD occurrence. While this approach does provide a cross-sectional overview, it cannot establish causality or serve as a predictive model.

To account for α-error inflation, *p*-values were adjusted using the Bonferroni–Holm correction [31]. The φ-coefficient was calculated to indicate an association between the dichotomous variables. Significance level α < 0.05 was chosen for all statistical analyses [32].

## 3. Results

A total of 45 cardiac centers across Germany participated in this study. Out of 287 completed questionnaires, the response rate was 58%. According to the German Association of Perfusion, an estimated 650 perfusionists are active in Germany [15]. Thus, the study sample represents 44% of the national perfusion workforce. Among the participants, 25.1% (n = 72) were female, while 74.9% (n = 215) were male. The mean age was 42.6 ± 11.9 years with an average professional experience of 13.5 ± 10.9 years. The mean BMI was 26.0 ± 3.7 kg/m^2^ (Table 1). Most participants were right-handed (89.5%; n = 257), followed by left-handed (5.6%; n = 16), and ambidextrous (4.9%; n = 14).

A strong positive correlation was observed between age and YoE (*p* < 0.001; r = 0.868). Age also showed a weak positive correlation with BMI (*p* = 0.038; r = 0.123).

**Figure 1:**
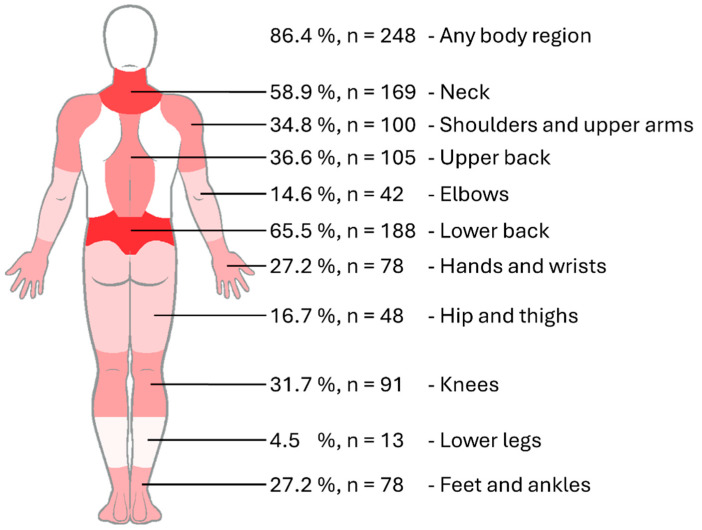
Annual prevalence of MSDs among German perfusionists (n = 287) across 10 body regions. Red shading illustrates prevalence proportionally, with greater saturation indicating higher rates.

Overall, 86.4% of participants reported experiencing MSDs in at least one body region within the previous 12 months (Figure 1). The lower back was the most frequently affected body region (65.5%), followed by the neck (58.9%). More than one-third of respondents reported complaints in the upper back (36.6%), shoulders and upper arms (34.8%), and knees (31.7%). Prevalence in the hands, wrists, feet, and ankles was reported as 27.2%. Annual prevalence rates for the hips, thighs, elbows, forearms, and lower legs were reported below 20%.

The distribution of perfusionists in the study cohort according to age, BMI, and YoE is presented in Table 2. For each characteristic, the table depicts the number and percentage of participants within each subgroup.

The exploratory subgroup analyses were performed utilizing chi-squared tests. Significant differences in MSD prevalence were observed across seven of the ten body regions between the age, BMI, and YoE groups. The results of these tests are listed in Table 3. Groups with no significant associations (*p* > 0.05) are not depicted.

There were no significant differences in the occurrence of MSDs between the genders in the chi-squared analysis. Regarding BMI, significant differences in prevalence rates of MSDs were observed only in the feet and ankles, with overweight participants reporting a higher prevalence, whereas normal-weight participants reported a lower prevalence (Table 3). More than 20 YoE was associated with an increased prevalence of MSDs in four body regions. Two of these regions, along with the lower back, presented significantly fewer MSDs in perfusionists with less professional experience (≤5 YoE). Older perfusionists (>55 years of age) consistently reported higher MSD prevalence rates with significant differences in six body regions. Four of these regions showed significantly lower MSD rates among younger participants (≤35 years). No significant associations were identified regarding MSDs in the elbow and forearm, upper back, or lower leg body regions.

## 4. Discussion

This nationwide cross-sectional study revealed a high prevalence of MSDs among perfusionists in Germany, with 86% reporting symptoms in at least one body region. This rate is comparable to those reported for other healthcare professionals, which range from 57% to 93% [5,8,9,10]. Therefore, perfusion staff appear to face a similar occupational risk.

The weak correlation between age and BMI (r = 0.123) provides insights into the demographic profile of perfusionists in Germany. While this suggests BMI as a risk factor for developing MSDs, it may be localized to weight-bearing regions, such as the feet and ankles, rather than representing a generalized risk factor. As this is the first nationwide assessment of MSDs in perfusionists, even small effects contribute to establishing baseline information. Further studies are needed to explore the broader significance for occupational health, both locally and globally.

**Age** has been considered a non-modifiable risk factor for MSD development [33]. Therefore, it can be assumed that prevalence rates are associated with age, even without occupational overexposure. Older age has previously been associated with increased MSD prevalence in the neck, shoulders, lower back, and knees [9,10,27,34,35]. In this study, age was the factor most consistently associated with the occurrence of MSDs. Perfusionists aged >55 years showed significantly higher prevalence rates (*p* < 0.05) in the neck, shoulders, hands, wrists, lower back, hip, thighs, and knees. The age group reported approximately 20% higher prevalence of MSDs in these body regions compared to the rest of the cohort. Age was associated with MSD prevalence in perfusionists and should be considered when assessing occupational risks. Because YoE and age were strongly correlated, the resulting collinearity prevented the use of multivariate models. Consequently, distinguishing between their individual effects remains challenging. As a result, potential risk factors identified in other healthcare professions have been evaluated for their applicability to perfusion staff, with age considered as an independent factor.

The highest prevalence of MSDs was reported in the **lower back** (65.5%). This finding connects with known risk factors for developing lower back pain such as prolonged static postures, non-neutral trunk positions, and handling of heavy or specialized equipment [5,8,9,25,26,29,34,35,36]. Based on the authors’ knowledge of the profession, these exposures are assumed to be present daily in perfusion and could contribute to the development of lower back complaints over the course of a career. Typical tasks of perfusionists include moving the HLM to and from the operating room, working in seated positions with none, limited or non-adjustable back support, and assuming forward-flexed or reaching postures to access sensors, tubing, and controls. Depending on the configuration of the HLM, perfusionists may also be required to lean downward while standing to operate equipment. Although these specific exposures were not formally recorded, they represent widely recognized aspects of routine perfusion practice. Significantly fewer complaints were observed among participants with ≤5 YoE, suggesting that lower back MSDs were developed only after some years in perfusion, potentially reflecting cumulative exposure to occupational conditions. A connection between professional experience and MSDs has been documented in other healthcare professions, especially with a sharp increase during the early years of employment [10,12,29,30].

The **neck** had a high prevalence of MSDs among the participants (58.9%). This may be due to frequent twisting, repetitive movements, and prolonged static position [5,8,9,10]. The prevalence was highest among older participants. However, a notable prevalence was also observed among younger perfusionists. Observations suggest that these rates may be linked to present occupational risks, such as frequent screen monitoring in unfavorable OR layouts and repeated reaching for or adjusting equipment.

MSDs in the **shoulders and upper arms** (34.8%) were significantly associated with increasing age and YoE. The highest subgroup prevalence was observed in participants with >20 YoE. Commonly reported risk factors for shoulder discomfort include repetitive use of the upper extremities, working with elevated arms, and handling tools [5,8,9,25,34]. As these characteristics seem common in perfusion practice, cumulative exposure may contribute to the observed patterns.

**Knee** complaints (31.7%) were also significantly associated with older age and higher YoE. Risk factors for the development of knee pain, identified in the literature, include prolonged standing, sustained static postures, increased YoE, and age [8,9,27,34]. As standing for extended hours in the OR is suggested to be inherent to perfusion, systemic occupational risk could be a possibility.

**Hand and wrist** complaints (27.2%) were significantly associated with older age. Although no further risk factors were identified in the present study, previous research on hemodialysis nurses has demonstrated an increased risk associated with daily assembly and dismantling of single-use components [37,38]. Because setup and disassembly of the HLM are key elements of perfusionists’ daily tasks, systematic occupational exposure cannot be ruled out and should be studied further.

Overweight participants had a significantly higher prevalence of symptoms in the **feet and ankles** (27.2%), indicating that weight-bearing stress may be a primary contributing factor [9]. Higher YoE was also associated with MSDs in the feet and ankles, which may indicate potential occupational risks [8,9].

**Hip and thigh** discomfort (16.7%) was associated with increased age and higher YoE. Prolonged standing is known to contribute to MSDs [5,25]. However, age is also correlated with reduced flexibility and MSDs in general [10,25,27]. The relatively low prevalence among younger participants suggests that these symptoms may be more age-related than occupation-specific, indicating that targeted interventions are of lesser urgency.

The prevalence of **upper back** complaints (36.6%) was high compared to other body regions. Despite this, the association did not reach statistical significance and is not well-reported in the literature. Complaints in the **elbows** (14.6%) and **lower legs** (4.5%) were comparatively low and showed no significant subgroup associations. While these regions appear to be less systematically affected, they may still merit continued observation in future studies.

### Limitations

This study has several limitations that should be considered when interpreting the results. The use of a self-administered questionnaire may have introduced recall bias as participants reported MSDs experienced over the past 12 months. Self-assessment also bears the potential for overestimation or underestimation of prevalence. These limitations were addressed, in part, by utilizing the validated and widely used NMQ.

The use of repeated statistical tests for each characteristic increases the risk of α-inflation. Although *p*-values were adjusted using the Bonferroni-Holm correction, this limitation necessitates cautious interpretation of the results. Additionally, the strong correlation between age and YoE introduced collinearity, making independent analyses challenging. Future studies should address this using improved data collection strategies.

Furthermore, the inability to apply multivariate regression because of collinearity and confounding risks limits the depth of the analysis. Although chi-square tests were used, the univariate approach restricts the exploration of complex connections and interactions. Subsequent research should adopt methods to overcome these constraints and provide better insights into MSD determinants among perfusionists.

The study design did not link the participants to specific cardiac centers or collect data on workplace conditions, such as specific work tasks, equipment, or work schedules. Additionally, MSD severity and duration, participation in training programs, and lifestyle activities were not recorded, limiting the assessment and possibility of correlations.

Finally, as this was the first nationwide survey of MSD prevalence among perfusionists in Germany, comparisons with previous studies were limited. Therefore, future studies should incorporate ergonomic workplace assessments, direct observations, and movement analyses to identify causal risk factors and support the development of targeted interventions.

## 5. Conclusions

This nationwide study revealed a high prevalence of MSDs among perfusionists in Germany, with 86% of respondents reporting symptoms in at least one body region within the last 12 months. Age and years of professional experience were considered as potential risk factors for developing MSDs. Occupational hazards previously described in other healthcare professions, such as prolonged static postures, non-neutral working positions, and equipment handling, may contribute to the development of MSDs among perfusionists. The lower back and neck were affected in more than half of the participants of this study and were previously associated with the aforementioned risk factors in other healthcare professions. Body regions with moderate to low prevalence in this cohort (shoulders, upper back, hands, knees, hips, and ankles) warrant further investigation, as occupational overexposure cannot be ruled out. Descriptive analyses exploring how MSD prevalence rates evolve throughout the professional career are also encouraged in future work. Efforts to assess and reduce rates of MSD occurrence in perfusion should include comprehensive ergonomic assessments, followed by targeted optimization of equipment, consideration of workload management, and training on postures. Implementing such measures may support long-term musculoskeletal health and improve occupational safety for perfusionists. As the healthcare system is facing workforce shortages, safeguarding the health of highly specialized perfusionists is crucial to guarantee adequate medical care for the entire population.

## Figures and Tables

**Table 1:** Demographic data of the perfusionists participating in the study. CI: confidence interval; SD: standard deviation.

Variable	Mean [95% CI]	SD (Range)
Age (years)	42.6 [41.3; 44.0]	11.9 (22–69)
BMI (kg/m^2^)	26.0 [25.6; 26.5]	3.7 (17.6–45.5)
Experience as perfusionist (years)	13.5 [12.2; 14.7]	10.9 (1–39)

**Table 2:** Distribution of participants within subgroups by age, BMI, and years of experience in perfusion (YoE), with the number of participants and percentage relative to the total cohort.

Age Groups	n	%	BMI Groups	n	%	Experience Groups	n	%
≤35 years	105	36.5	Underweight	2	0.7	≤5 YoE	102	35.5
36–45 years	68	23.7	Normal weight	126	43.9	6–10 YoE	48	16.7
46–55 years	53	18.5	Overweight	124	43.2	11–20 YoE	56	19.5
>55 years	61	21.3	Obese	35	12.2	>20 YoE	81	28.2

**Table 3:** Significant differences in MSD prevalence by group, associated body region, and subgroup-specific versus rest of the cohort prevalences. Depicted are adjusted *p*-values after Bonferroni–Holm correction and φ-coefficients.

Group (vs. Rest)	Body Region	MSD Prev. (Group vs. Rest)			*p*	φ
Normal weight	Feet and ankles	20%	vs.	33%	0.039	−0.146
Overweight	Feet and ankles	36%	vs.	21%	0.024	0.163
YoE ≤ 5	Shoulder and upper arms	24%	vs.	41%	0.009	−0.176
	Lower Back	55%	vs.	71%	0.02	−0.166
	Knees	21%	vs.	38%	0.009	−0.177
YoE > 20	Shoulder and upper arms	53%	vs.	28%	0.004	0.24
	Hip and thighs	26%	vs.	13%	0.036	0.155
	Knees	49%	vs.	25%	0.004	0.238
	Feet and ankles	38%	vs.	23%	0.032	0.156
Age ≤ 35	Shoulder and upper arms	24%	vs.	41%	0.009	−0.176
	Lower Back	54%	vs.	72%	0.008	−0.179
	Hip and thighs	9%	vs.	21%	0.015	−0.166
	Knees	14%	vs.	42%	0.004	−0.284
Age > 55	Neck	75%	vs.	54%	0.012	0.174
	Shoulder and upper arms	54%	vs.	30%	0.004	0.21
	Hands and wrists	46%	vs.	22%	0.004	0.219
	Lower Back	79%	vs.	62%	0.045	0.144
	Hip and thighs	33%	vs.	12%	0.005	0.224
	Knees	49%	vs.	27%	0.004	0.195

## Data Availability

The raw data supporting the conclusions of this study will be made available by the authors upon request.

## Abbreviations

The following abbreviations are used in this manuscript:

MSD	Musculoskeletal disorder
NMQ	Nordic Musculoskeletal Questionnaire
HLM	Heart–lung machine
OR	Operating room
YoE	Years of experience
BMI	Body mass index
CI	Confidence interval
SD	Standard deviation

## References

[1] National Academies of Sciences, Engineering, and Medicine (2020). Selected Health Conditions and Likelihood of Improvement with Treatment.

[2] de Kok J., Vroonhof P., Snijders J., Roullis G., Clarke M. (2019). Work-Related MSDs Prevalence Costs and Demographics in the EU.

[3] Da Costa B.R., Vieira E.R. (2010). Risk factors for work-related musculoskeletal disorders: A systematic review of recent longitudinal studies. Am. J. Ind. Med..

[4] Dauber T., Isusi I. Work-Related Musculoskeletal Disorders: Prevalence, Costs and Demographics in the EU: National Report: Germany. https://osha.europa.eu/sites/default/files/work_related_MSDs_Germany.pdf.

[5] Jacquier-Bret J., Gorce P. (2023). Prevalence of body area work-related musculoskeletal disorders among healthcare professionals: A systematic review. Int. J. Environ. Res. Public Health.

[6] Skela-Savič B., Pesjak K., Hvalič-Touzery S. (2017). Low back pain among nurses in Slovenian hospitals: Cross-sectional study. Int. Nurs. Rev..

[7] Davis K.G., Kotowski S.E. (2015). Prevalence of musculoskeletal disorders for nurses in hospitals, long-term care facilities, and home health care: A comprehensive review. Hum. Factors.

[8] Suganthirababu P., Parveen A., Mohan Krishna P., Sivaram B., Kumaresan A., Srinivasan V., Vishnuram S., Alagesan J., Prathap L. (2023). Prevalence of work-related musculoskeletal disorders among health care professionals: A systematic review. Work.

[9] Sun W., Yin L., Zhang T., Zhang H., Zhang R., Cai W. (2023). Prevalence of work-related musculoskeletal disorders among nurses: A meta-analysis. Iran. J. Public Health.

[10] Tavakkol R., Karimi A., Hassanipour S., Gharahzadeh A., Fayzi R. (2020). A Multidisciplinary focus review of musculoskeletal disorders among operating room personnel. J. Multidiscip. Healthc..

[11] Heller J., Melzer S. Berufsbild. https://perfusiologie.de/ueber-uns/berufsbild/.

[12] Alwabli Y., Almatroudi M.A., Alharbi M.A., Alharbi M.Y., Alreshood S., Althwiny F.A. (2020). Work-related musculoskeletal disorders among medical practitioners in the hospitals of Al’Qassim Region, Saudi Arabia. Cureus.

[13] Bernal D., Campos-Serna J., Tobias A., Vargas-Prada S., Benavides F.G., Serra C. (2015). Work-related psychosocial risk factors and musculoskeletal disorders in hospital nurses and nursing aides: A systematic review and meta-analysis. Int. J. Nurs. Stud..

[14] Thacker H., Yasobant S., Viramgami A., Saha S. (2023). Prevalence and determinants of (work-related) musculoskeletal disorders among dentists—A cross sectional evaluative study. Indian J. Dent. Res..

[15] Heller J., Melzer S. Herzzentren. https://perfusiologie.de/karriere/herzzentren/.

[16] DIE WELT. Kardiotechniker: Die Verkannten Lebensretter im OP—WELT. https://www.welt.de/gesundheit/article168921312/Diese-Maenner-sind-die-verkannten-Lebensretter-im-OP.html.

[17] Debeuckelaere G., Klüß C., Ruck K., Nagaraj N.G., Brajlović E., Kjellberg G., Talmaciu C., Lenart Z., Muraskauskaite M., Ristić N. (2025). Perfusion education and training in Europe anno. Perfusion.

[18] Riley W. Milwaukee, Wisconsin, 2025, 2024 [Annual Report]. https://www.abcp.org/UserFiles/2024AnnualReport.pdf.

[19] Turnage C., DeLaney E., Kulat B., Guercio A., Palmer D., Ann Rosenberg C., Spear K., Boyne D., Johnson C., Riley W.A. (2017). A 2015–2016 Survey of American Board of cardiovascular Perfusion Certified Clinical Perfusionists: Perfusion profile and clinical trends. J. Extra Corpor. Technol..

[20] Lewis D.M., Dove S., Jordan R.E. (2016). Results of the 2015 Perfusionist salary study. J. Extra Corpor. Technol..

[21] Kuorinka I., Jonsson B., Kilbom A., Vinterberg H., Biering-Sørensen F., Andersson G., Jørgensen K. (1987). Standardised Nordic questionnaires for the analysis of musculoskeletal symptoms. Appl. Ergon..

[22] Liebers F., Freyer M., Freitag S., Dulon M., Hegewald J., Latza U. (2022). Fragebogen zu Muskel-Skelett-Beschwerden (FB*MSB).

[23] Kreis L., Liebers F., Dulon M., Freitag S., Latza U. (2021). Verwendung des nordischen Fragebogens zu Muskel-Skelett-Beschwerden. Zentralblatt Arbeitsmed..

[24] Liebers F., Freyer M., Dulon M., Freitag S., Michaelis M., Latza U., Hegewald J. (2024). Neuer deutschsprachiger Fragebogen zur standardisierten Erfassung von Muskel-Skelett-Beschwerden im Betrieb. Zentralblatt Arbeitsmed..

[25] Passali C., Maniopoulou D., Apostolakis I., Varlamis I. (2018). Work-related musculoskeletal disorders among Greek hospital nursing professionals: A cross-sectional observational study. Work.

[26] Bin Homaid M., Abdelmoety D., Alshareef W., Alghamdi A., Alhozali F., Alfahmi N., Hafiz W., Alzahrani A., Elmorsy S. (2016). Prevalence and risk factors of low back pain among operation room staff at a Tertiary Care Center, Makkah, Saudi Arabia: A cross-sectional study. Ann. Occup. Environ. Med..

[27] Heidari M., Borujeni M.G., Rezaei P., Kabirian Abyaneh S. (2019). Work-related musculoskeletal disorders and their associated factors in nurses: A cross-sectional study in Iran. Malays. J. Med. Sci..

[28] Ribeiro T., Serranheira F., Loureiro H. (2017). Work related musculoskeletal disorders in primary health care nurses. Appl. Nurs. Res..

[29] Videman T., Ojajärvi A., Riihimäki H., Troup J.D.G. (2005). Low back pain among nurses: A follow-up beginning at entry to the nursing school. Spine.

[30] Mohseni-Bandpei M.A., Ahmad-Shirvani M., Golbabaei N., Behtash H., Shahinfar Z., Fernández-de-las-Peñas C. (2011). Prevalence and risk factors associated with low back pain in Iranian surgeons. J. Manip. Physiol. Ther..

[31] Hemmerich W.A. StatistikGuru: Rechner zur Adjustierung des α-Niveaus. https://statistikguru.de/rechner/adjustierung-des-alphaniveaus.html.

[32] Döring N. (2023). Forschungsmethoden und Evaluation in den Sozial- und Humanwissenschaften. Vollständig Überarbeitete, Aktualisierte und Erweiterte Auflage.

[33] Mody G.M., Brooks P.M. (2012). Improving musculoskeletal health: Global issues. Best Pract. Res. Clin. Rheumatol..

[34] Dong H., Zhang Q., Liu G., Shao T., Xu Y. (2019). Prevalence and associated factors of musculoskeletal disorders among Chinese healthcare professionals working in tertiary hospitals: A cross-sectional study. BMC Musculoskelet. Disord..

[35] Karahan A., Kav S., Abbasoglu A., Dogan N. (2009). Low back pain: Prevalence and associated risk factors among hospital staff. J. Adv. Nurs..

[36] Nourollahi M., Afshari D., Dianat I. (2018). Awkward trunk postures and their relationship with low back pain in hospital nurses. Work.

[37] Ibenthal E., Hinricher N., Nienhaus A., Backhaus C. (2024). Hand and wrist complaints in dialysis nurses in Germany: A survey of prevalence, severity, and occupational associations. Ann. Work. Expo. Health.

[38] Westergren E., Lindberg M. (2022). Work-related musculoskeletal complaints among haemodialysis nurses: An exploratory study of the work situation from an ergonomic perspective. Work.

